# Five-Year Follow-Up of an Eight-Year-Old Boy With a Scleral-Fixated Carlevale Intraocular Lens After Ocular Trauma

**DOI:** 10.7759/cureus.114849

**Published:** 2026-08-20

**Authors:** Zoi Drakou, Stratos Gotzaridis, Agathi Kouri, Eirini Maliagkani

**Affiliations:** 1 Department of Ophthalmology, Ofthalmiatreio Athinon, Athens, GRC; 2 Department of Ophthalmology, My Retina Athens Eye Center, Athens, GRC; 3 Department of Ophthalmology, A. Kyriakou Children's Hospital Athens, Athens, GRC; 4 1st Department of Ophthalmology, National and Kapodistrian University of Athens, Athens, GRC

**Keywords:** carlevale iol, pars plana vitrectomy, pediatric ocular trauma, scleral fixation, traumatic aphakia, visual rehabilitation

## Abstract

Management of pediatric aphakia without capsular support remains challenging, particularly following ocular trauma. Long-term outcomes of sutureless scleral fixation of the Carlevale intraocular lens (IOL) in children remain limited. We present the surgical management and five-year follow-up of a child with traumatic aphakia treated with a Carlevale IOL.

An eight-year-old boy sustained a penetrating ocular injury caused by a knife, resulting in a full-thickness corneal laceration, traumatic cataract, and inadequate capsular support. Following primary corneal repair and lens removal elsewhere, the patient was referred to our clinic. A 25-gauge pars plana vitrectomy and sutureless scleral fixation of a Carlevale IOL were performed. At the five-year follow-up examination, the IOL remained well centered without tilt, haptic erosion, or significant inflammatory complications. Visual acuity improved progressively, reaching 0.0 LogMAR without refractive correction at the final examination.

This case demonstrates a favorable anatomical and visual outcome at five years following sutureless scleral fixation of a Carlevale IOL in a child with traumatic aphakia and inadequate capsular support. The IOL remained stable at the five-year examination, with excellent uncorrected visual acuity and a favorable refractive outcome. This case suggests a potential role for the Carlevale IOL in the management of pediatric traumatic aphakia.

## Introduction

Pediatric aphakia without adequate capsular support for stable intraocular lens (IOL) fixation remains a challenging condition, particularly following ocular trauma. Visual rehabilitation is complicated by ongoing ocular growth, difficulties in IOL power calculation, and the risk of amblyopia [1-3].

Several surgical techniques have been proposed for eyes lacking capsular support, including anterior chamber (AC), iris-fixated, and scleral-fixated IOLs, each associated with specific limitations, including potential endothelial, iris-related, or fixation-related complications [1-4]. The Carlevale IOL (Soleko IOL Division, Italy) is a foldable posterior chamber IOL designed for sutureless scleral fixation using T-shaped haptics that are externalized and anchored within the sclera. It has demonstrated favorable visual outcomes and encouraging anatomical stability in adults, with an acceptable safety profile [5,6].

Experience with the Carlevale IOL in children remains limited, particularly after ocular trauma, and long-term follow-up data are scarce [2,4-6]. We report the five-year anatomical and visual outcomes of a Carlevale IOL in a child with traumatic aphakia and inadequate capsular support. To our knowledge, this is the first reported pediatric case of a Carlevale IOL implantation following ocular trauma with such an extended follow-up period.

## Case presentation

An eight-year-old boy presented to the Emergency Department with a penetrating left eye injury caused by a knife. Initial visual acuity was counting fingers (CF). Slit-lamp biomicroscopy revealed a C-shaped full-thickness corneal laceration (2-7 o’clock), shallow AC (Seidel sign positive), and post-traumatic cataract, with no retinal pathology on fundoscopy. Primary corneal repair was performed (seven 10/0 Nylon sutures), followed by partial lens aspiration through a 2.75 mm incision.

**Figure 1:**
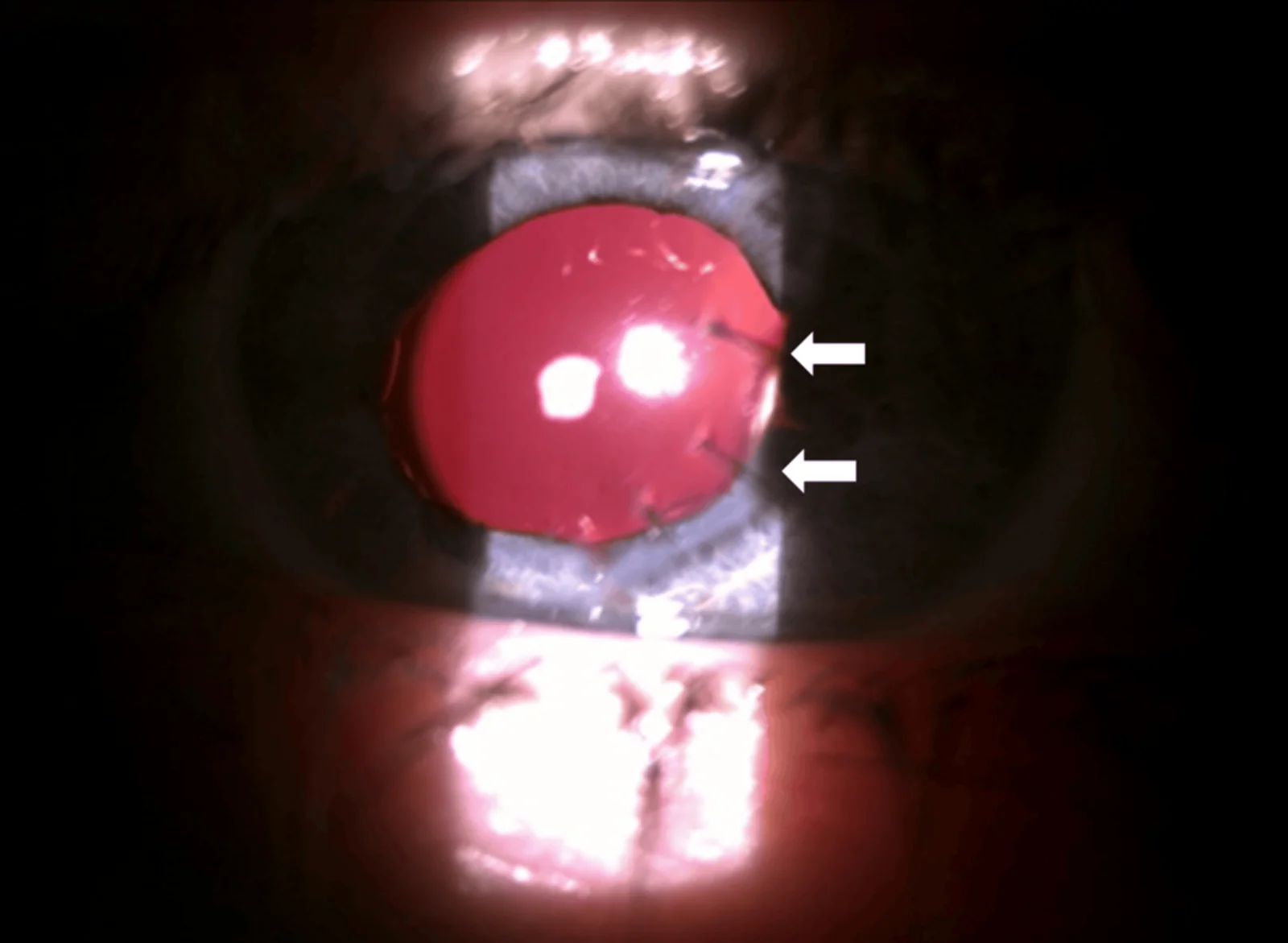
Slit-lamp photography of the left eye at first visit showing C-shaped corneal laceration and stitches in situ (white arrows).

Two months later, the patient was referred to our clinic with best-corrected visual acuity (BCVA) of CF at 50 cm, intraocular pressure (IOP) of 12 mmHg, and C-shaped corneal stitches in situ (Figure 1). The IOP of the right eye was also 12 mmHg. Fundoscopy revealed no retinal pathology. Examination revealed residual capsular material with insufficient zonular support for conventional in-the-bag or sulcus IOL implantation. Therefore, implantation of a scleral-fixated Carlevale IOL was planned. As reliable biometry of the injured eye was not feasible in the presence of corneal sutures following traumatic corneal repair, biometry of the fellow eye was used (axial length 22.76 mm, AC depth 3.49 mm, K1 7.94 mm, K2 7.59 mm); IOL power was calculated using the Sanders-Retzlaff-Kraff (SRK)/II formula (A-constant 118.5), yielding a 22.0 D lens targeting emmetropia in adulthood.

The patient underwent 25-gauge three-port pars plana vitrectomy (PPV) with induced posterior vitreous detachment (PVD). PVD induction was technically challenging due to the patient's young age. The remaining capsule and lens material were removed with the vitreous cutter. The Carlevale IOL was inserted through a 2.75 mm corneal tunnel, and the T-shaped haptics were secured within scleral flaps 2.5 mm posterior to the limbus at three and nine o’clock. No intraoperative retinal pathology was identified. A single 10-0 Nylon corneal stitch was placed at 11 o’clock in the sclerocorneal junction. At the end of the surgery, the trocars and infusion cannula were removed, and cefuroxime (5 mg) and dexamethasone (2 mg) were administered subconjunctivally.

**Figure 2:**
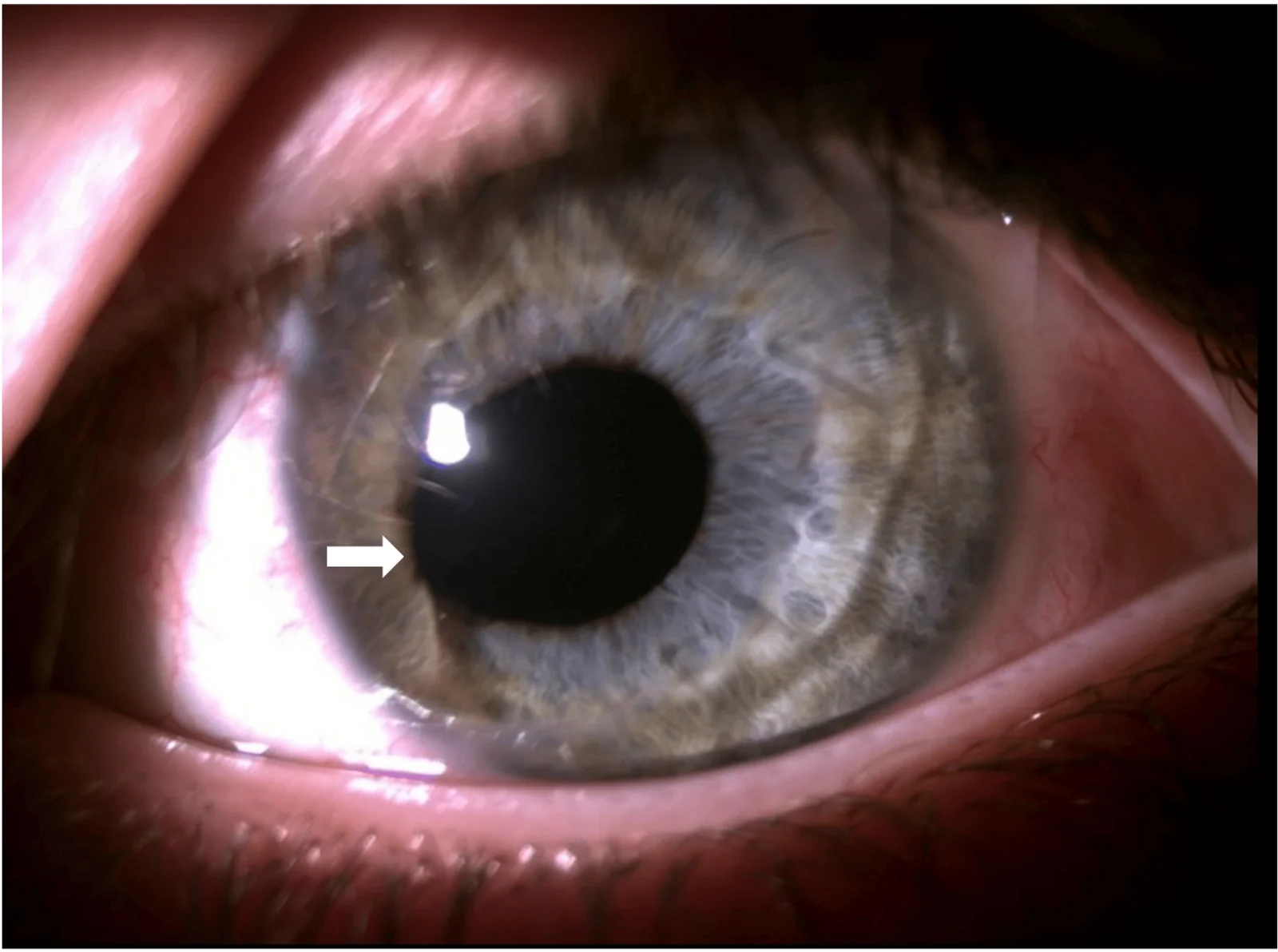
Slit-lamp photography of the left eye at eight months after surgery showing a centered Carlevale intraocular lens (IOL) and mild nasal iris ectopia (white arrow).

**Figure 3:**
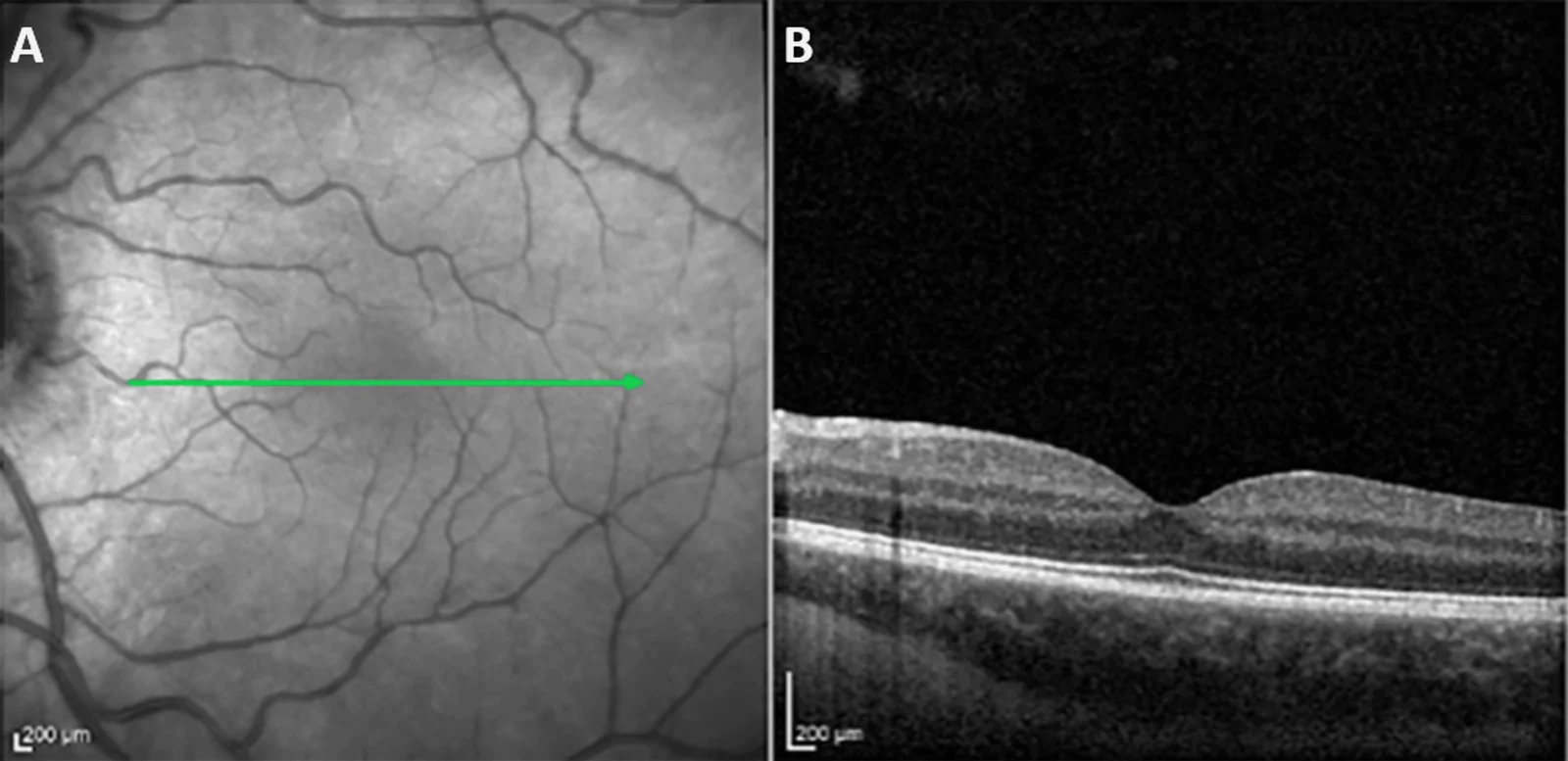
(A, B) Macular optical coherence tomography (OCT) one year after surgery demonstrating preserved foveal contour without macular pathology.

Visual recovery was progressive. BCVA improved from 1.1 LogMAR on postoperative day 1 to 0.15 LogMAR at one year with spectacle correction (Table 1). Original corneal sutures were removed four months after surgery. Amblyopia therapy (two hours daily patching of the fellow eye) was recommended, though compliance could not be confirmed. At the eight-month follow-up, slit-lamp examination demonstrated a well-centered Carlevale IOL with mild nasal iris ectopia (Figure 2), while macular optical coherence tomography (OCT) remained normal one year postoperatively (Figure 3). The patient was subsequently lost to follow-up for approximately three years.

**Table 1:** Summary of key clinical findings and outcomes of the left eye at each follow-up visit. AS-OCT: anterior segment optical coherence tomography; BCVA: best-corrected visual acuity; CF: counting fingers; PH: pinhole; sph: sphere; OCT: optical coherence tomography; IOL: intraocular lens; IOP: intraocular pressure

Time	BCVA (LogMAR)	Refraction	IOP (mmHg)	Key clinical findings
Preoperative	CF (50 cm)	-	12	Traumatic aphakia, post-traumatic cataract, C-shaped corneal scar with sutures; retina attached
Postoperative				
Day 1	1.1 (0.7 PH)	+7.50 − 2.50 × 97°	3	Retina attached; no postoperative complications
10 days	0.2	+4.50 − 2.75 × 90°	10	Stable postoperative course
1 month	0.2	+4.00 − 3.00 × 80°	11	Corneoscleral suture removed
4 months	0.2	+4.25 − 1.75 × 100°	11	Initial corneal sutures removed
8 months	0.15	+2.00 − 0.25 × 120°	12	Well-centered Carlevale IOL; mild nasal iris ectopia
1 year	0.15	+1.50 sph	11	Normal macular OCT; amblyopia therapy (2-hour daily patching) advised
5 years	0.0	−1.25 × 50°	11	Stable IOL without tilt, haptic capture, conjunctival erosion, or inflammation; nasal iris cyst and temporal cornea-iridal synechia on AS-OCT; normal macular OCT; peripheral inferotemporal pigmented demarcation line without retinal tear or subretinal fluid

**Figure 4:**
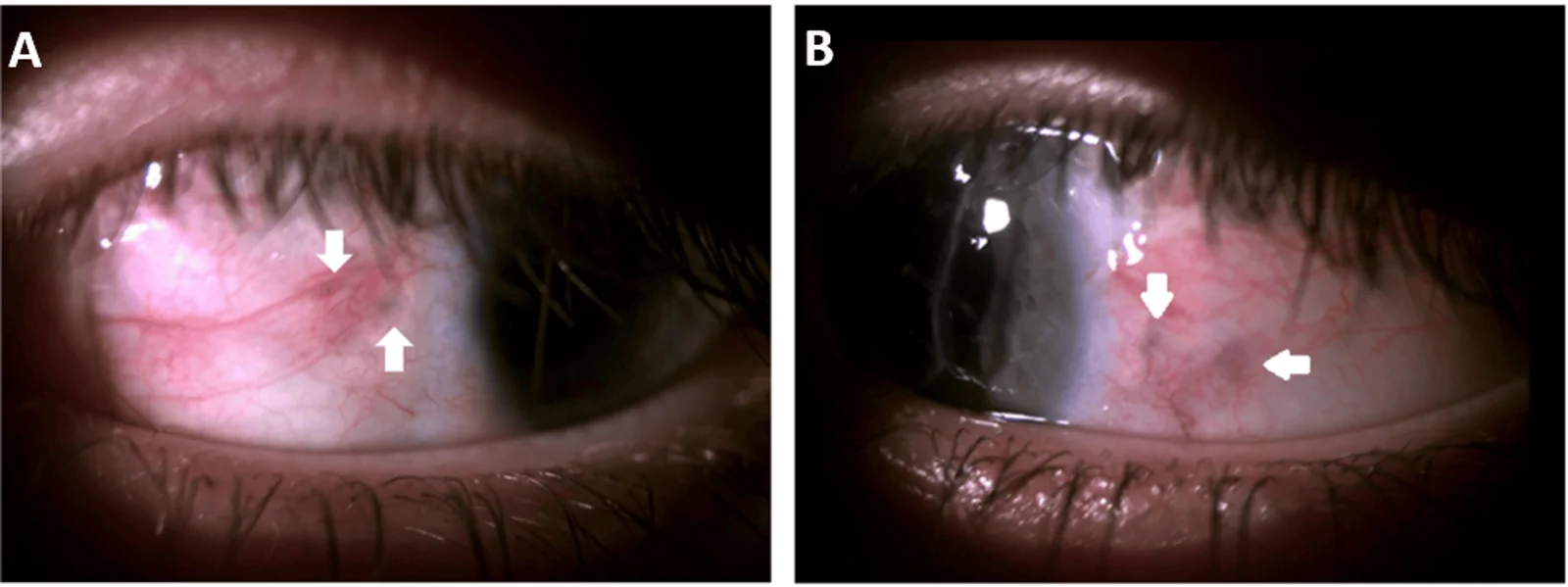
(A, B) Slit-lamp photography of the left eye at five years showing stable scleral fixation. White arrows indicate the haptic fixation sites, with no evidence of haptic capture, conjunctival inflammation, or erosion.

**Figure 5:**
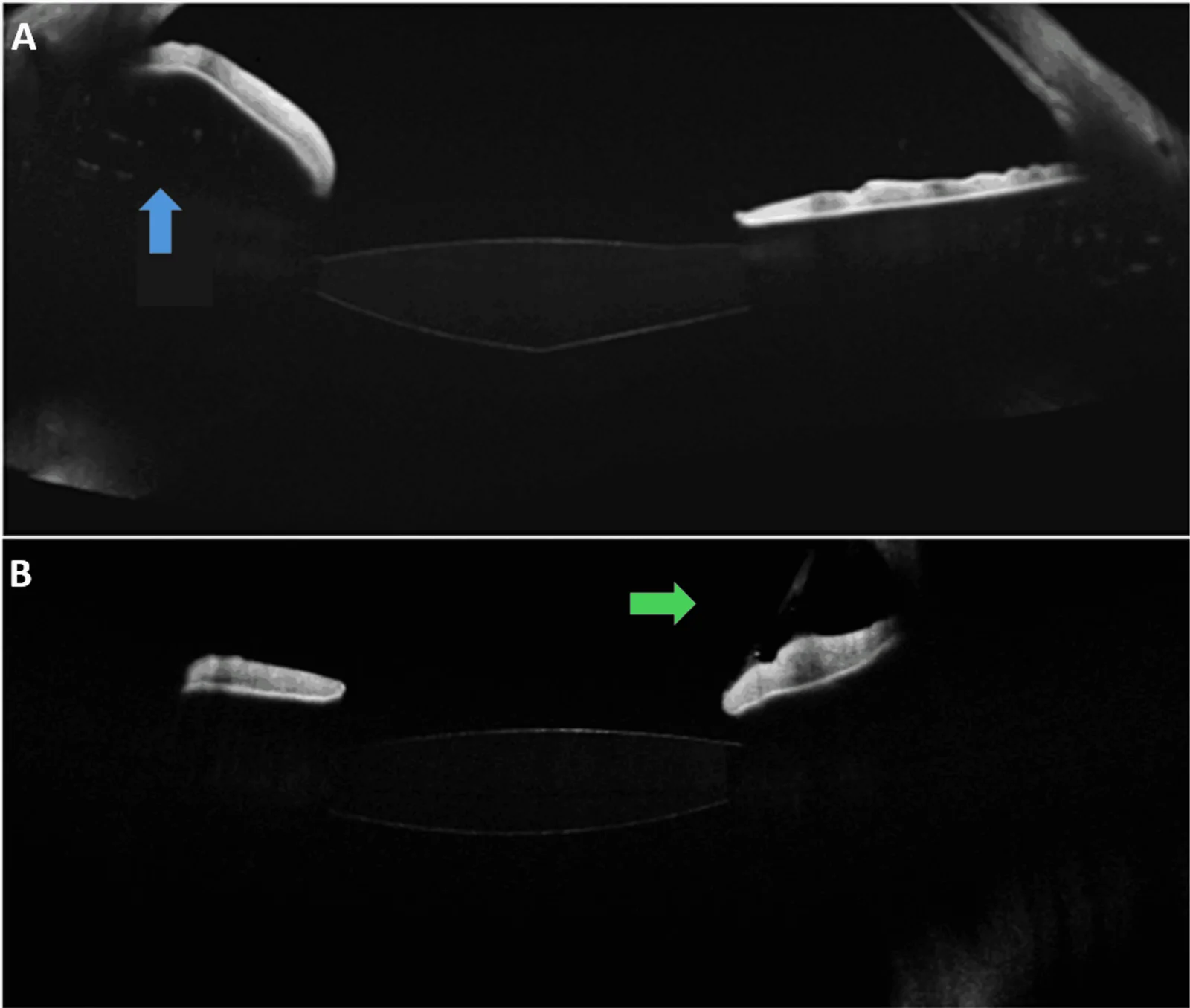
(A, B) Anterior segment optical coherence tomography (AS-OCT) demonstrating a nasal iris cyst (blue arrow) and temporal post-traumatic cornea-iridal synechia (green arrow).

**Figure 6:**
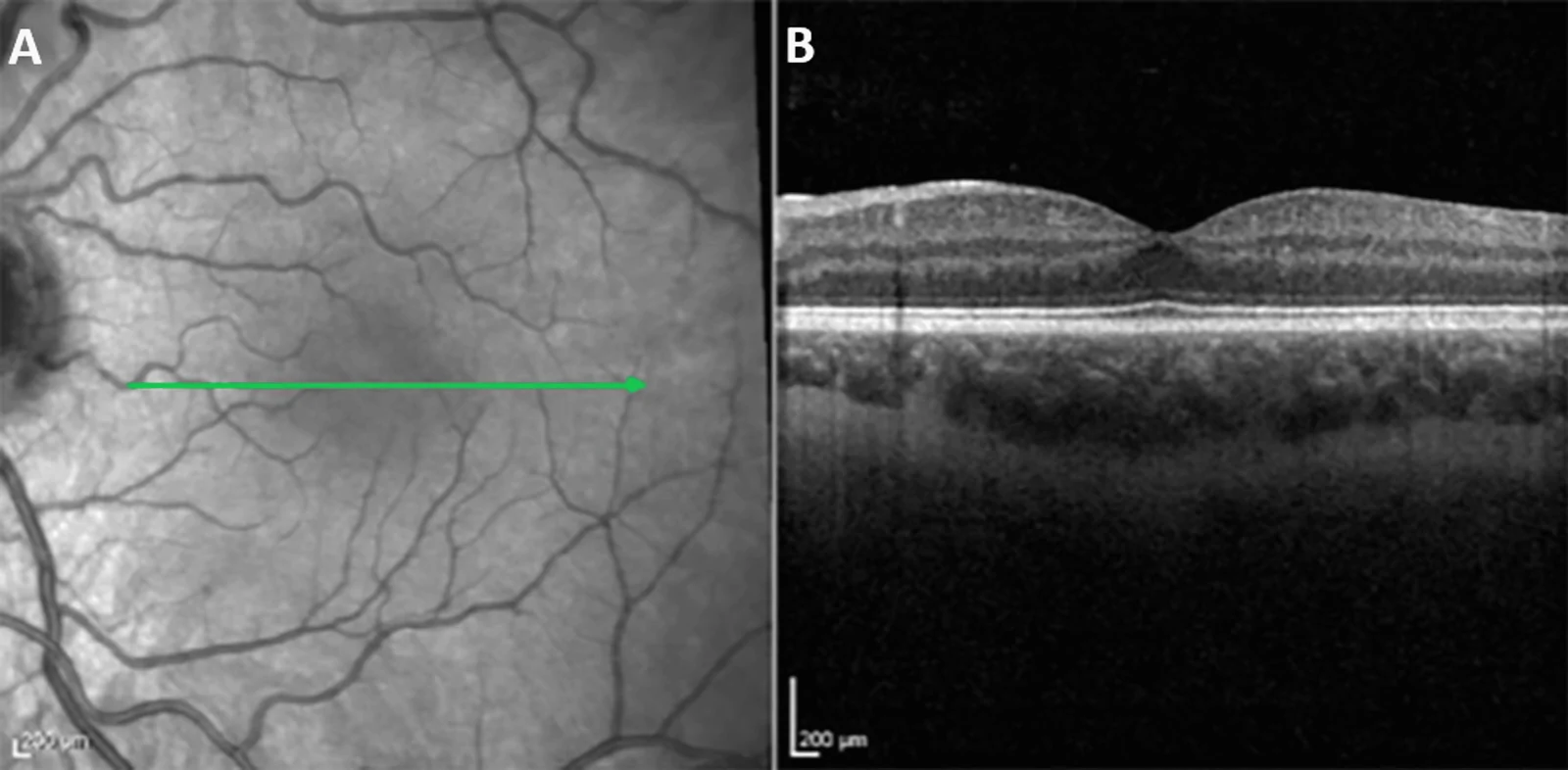
(A, B) Macular optical coherence tomography (OCT) five years after surgery demonstrating preserved foveal contour without macular pathology.

At the five-year visit, uncorrected visual acuity of the left eye was 0.0 LogMAR (Table 1), and IOP was 11 mmHg in the left eye and 12 mmHg in the right eye. The Carlevale IOL remained well-centered with no tilt, haptic capture, or conjunctival erosions (Figure 4). Anterior segment OCT (AS-OCT) revealed a nasal iris cyst and temporal cornea-iris synechiae (Figure 5). Fundoscopy showed a peripheral inferotemporal pigmented demarcation line without subretinal fluid or retinal tear. Macular OCT was normal (Figure 6). No further surgical intervention was required.

## Discussion

The Carlevale IOL has demonstrated favorable visual outcomes in adult aphakia without adequate capsular support, with an acceptable safety profile [2,5]. Ferrer-Alapont et al., in a cohort of 268 patients, reported a 26.6% cumulative probability of postoperative macular edema at 12 months and a 1.5% incidence of retinal detachment, while haptic rupture or IOL disenclavation occurred in 3.0% of eyes [6]. In a series of 169 adult eyes undergoing Carlevale IOL implantation combined with three-port PPV, Georgalas et al. reported significant visual improvement and stable IOL positioning, with no cases of IOL capture or need for reoperation [5].

Despite these encouraging adult outcomes, management of pediatric aphakia without capsular support remains challenging. Although optical correction with contact lenses or spectacles is possible, poor compliance and the risk of amblyopia often favor secondary IOL implantation [1,2]. Available fixation strategies - including AC, iris-fixated, and scleral-fixated IOLs - each carry specific limitations and complications, and no single approach has emerged as the standard of care [3,4]. The Carlevale IOL was selected in our case because its posterior chamber, scleral-fixated design avoids iris fixation and AC placement, thereby reducing concerns related to iris chafing and endothelial damage associated with these alternative approaches. Furthermore, it provides sutureless scleral fixation, with favorable stability outcomes reported in adults. Recent reports have demonstrated encouraging short-term outcomes with the Carlevale IOL in children, but long-term follow-up data remain scarce [2-4]. To our knowledge, the present case represents the longest reported follow-up of a Carlevale IOL implanted in a child following ocular trauma.

In our case, the additional complexity of ocular trauma further restricted available surgical options, as the corneal laceration and complete absence of capsular support excluded many alternatives. Biometry was performed on the fellow eye, targeting emmetropia in adulthood. This decision was supported by the emmetropic status of both the fellow eye and the parents, as well as evidence from Rauscher et al. demonstrating no statistically significant increase in axial length in male patients between 10 and 17 years of age [7]. A 22.0 D Carlevale IOL was selected accordingly.

Five years after surgery, the patient achieved an uncorrected visual acuity of 0.0 LogMAR. Clinical examination showed a well-centered IOL without evident tilt, haptic capture, conjunctival erosion, or significant inflammatory complications. The initial postoperative hyperopia gradually evolved toward emmetropia, resulting in an encouraging refractive outcome at the five-year follow-up [4,7]. Changes in astigmatic magnitude and axis during follow-up may also have been influenced by the previous corneal trauma, subsequent suture removal, and measurement variability. IOL tilt or decentration was considered less likely because the IOL remained well centered without evident tilt at the five-year examination. Notably, this favorable visual outcome was achieved despite uncertain compliance with the prescribed amblyopia patching regimen.

The peripheral inferotemporal pigmented demarcation line identified at the five-year visit may represent a previous asymptomatic retinal detachment. The technically demanding PVD induction during surgery may have contributed to this finding; however, its etiology remains uncertain, and a causal relationship cannot be established. Since no visible retinal tear or hole, subretinal fluid, or vitreoretinal traction was identified on fundus examination, and considering the inferior location of the lesion, the risk of progression was deemed low, and observation was considered the most appropriate management [8].

AS-OCT demonstrated a nasal iris cyst and temporal post-traumatic cornea-iridal synechia. These findings were clinically stable, were not associated with visual impairment, and did not require additional intervention. They were considered more likely related to the previous ocular trauma and associated inflammatory response than to the IOL itself [9].

The main limitation of this report is the absence of follow-up data between the first and fifth postoperative years, which precludes assessment of the intermediate clinical course. Additionally, the inability to perform biometry on the injured eye introduced inherent uncertainty into IOL power calculation, while the absence of repeat axial-length measurements precluded objective assessment of ocular growth during follow-up. Endothelial cell density measurements were also unavailable, limiting objective assessment of long-term corneal endothelial status.

## Conclusions

This case demonstrates a favorable five-year anatomical and visual outcome following sutureless scleral fixation of a Carlevale IOL in a child with traumatic aphakia and inadequate capsular support. The IOL remained stable at the five-year examination, with excellent uncorrected visual acuity and a favorable refractive outcome. Although larger pediatric studies with continuous long-term follow-up are needed, this case provides valuable evidence regarding the potential role of the Carlevale IOL in the management of pediatric traumatic aphakia.
